# Assessment of reliability and validity of specific agility test indicators for amateur boxing

**DOI:** 10.7717/peerj.21458

**Published:** 2026-06-24

**Authors:** Yifei Luo, Dexin Wang, Qingyuan Yang, Chao Chen, Rui Wu

**Affiliations:** 1 School of Athletic Performance, Shanghai University of Sport, Shanghai, China; 2 Shanghai Key Lab of Human Performance, Shanghai University of Sport, Shanghai, China; 3 Sports Science Research Center of Xinjiang Uygur Autonomous Region, Urumqi, China; 4 Dalian University College of Physical Education, Dalian University, Da Lian, Liao Ning, China

**Keywords:** Boxing, Combat sport, Elite athletes, Boxing-specific agility, Reliability and validity, Test validation, Athletic performance

## Abstract

Based on boxing’s unique traits and recent rule changes (*e.g*., the 2013 10-point system, removal of male headgear, and 2017 adjustments to women’s rounds), which heighten agility demands, we developed the Boxing-Specific Agility Test (BSAT). To examine BSAT’s reliability, validity, sensitivity, and its relationship with planned agility, sprinting, jumping, and eye-hand coordination, 26 elite male boxers (16 Chinese, 10 Cuban, including five world-class) were recruited. The mean age and training experience of the participants were equal to 22 ± 3.31 years old and 11.54 ± 2.34 years, respectively. In addition to the BSAT, participants underwent planned agility tests (T-test and Y pre-planned agility, YPA), reactive agility test (Y reactive agility, YRA), perceptual decision-making assessment (Decision-making time, DMT), jumping tests (Counter movement jump, CMJ and Squat-Jump SJ), sprint tests (6 and 10 m), and a eye-hand coordination (EHC) test. To determine the discriminant validity of the BSAT, participants were divided into two groups based on their performance in international and domestic competitions: the Top Elite Group (*n* = 12) and the General Elite Group (*n* = 14). The retest results collected at 7-day intervals demonstrated that the BSAT exhibits high reliability, validity, and sensitivity (ICC = 0.966, *p* < 0.001). It accurately evaluates the specialized agility qualities of boxers and can detect small yet meaningful changes in performance. Top elite athletes demonstrated significantly better BSAT performance than the general elite group, evidenced by shorter total time (30.85 ± 3.52 s *vs*. 37.97 ± 3.39 s; *p* < 0.001) and faster average response time (1.5 ± 0.23 s *vs*. 1.98 ± 0.23 s; *p* < 0.001). The test’s discriminative power was robust, with an Area Under the Curve (AUC) of 0.93 (95% CI [0.84–0.99]; *p* < 0.001). BSAT scores strongly correlated with both the planned agility T-test (r = 0.76–0.77, *p* < 0.001) and the reactive Y-test (r = 0.77, *p* < 0.001), and showed moderate to high correlations with other physical traits like sprinting (r = 0.49–0.50, *p* < 0.05), jumping (r = 0.53–0.65, *p* < 0.01), and eye-hand coordination (r = 0.70–0.72, *p* < 0.001). In conclusion, the BSAT is a reliable and valid tool for assessing boxing-specific agility. Additionally, the BSAT can serve as a practical tool for coaches to preliminarily assess boxers’ sprinting ability, lower-body power, and eye-hand coordination. The test also allows for real-time observation of sport-specific movements, helping to identify technical weaknesses.

## Introduction

Boxing is one of the oldest combat sports across human cultures, with a history potentially exceeding 4,000 years (Bianco et al., 2013; Chaabène et al., 2015). It first entered the ancient Olympic Games in 688 BC (Poliakoff, 1987; Sesé, 2008). In the history of the modern Olympics, boxing became an official event at the 3rd Games in St. Louis, USA, in 1904. A century later, women’s boxing was included at the 2012 London Olympics. Since boxing became an Olympic sport, its rules have undergone various modifications in line with the times. The most recent changes were implemented by the International Boxing Association in 2013: the scoring system shifted to the professional boxing 10-point must system, with five judges scoring based on established criteria, and headgear was abolished for male boxers (AIBA, 2015). In 2017, women’s bouts were changed from four 2 min rounds to three 3 min rounds (AIBA, 2021). Boxers must continuously execute offensive, defensive, and transitional technical actions within each 3-min round to secure victory. Particularly after the adoption of the 10-point system, bouts have become more intense (Davis et al., 2018). Therefore, boxing places significant physiological demands on athletes, requiring high level of muscular endurance, strength, power, and cardiovascular fitness (Uca & Kilinç, 2020; Zhong & Bu, 2022; Finlay et al., 2023; Wu et al., 2024a). Furthermore, agility is another physical attribute inherent to boxers (Sienkiewicz-Dianzenza & Maszczyk, 2019; Bu, 2022; Ji & Yoon, 2022; Niu et al., 2024).

Agility is defined as “a rapid whole-body movement that alters speed or direction in response to stimuli” (Sheppard & Young, 2006). It is associated with human strength, speed, balance, coordination, cognitive components, and decision-making (Fiorilli et al., 2017; Liefeith et al., 2018; Çakmakçı et al., 2019). Agility is crucial for combat sports, as it enables athletes to execute tactical maneuvers swiftly and comprehensively through maintaining dynamic balance, speed, and accuracy during multi-directional planar movement (Marković, Mišigoj-Duraković & Trninić, 2005). Boxing’s activity pattern is intermittent, characterized by short bursts of high-intensity exertion (Slimani et al., 2017). And its primary techniques involve highly dynamic, transient, and rapid movements (Chaabène et al., 2015). This intensity escalated further after the implementation of the 10-point scoring system. In a prior study, Davis et al. (2018) employed video analysis to reveal a 20% increase in boxers’ ring movement. The Activity Rate per Round in Actions per Second (APS)—encompassing all offensive and defensive actions—progressively increased from the first to the second and third rounds (Davis et al., 2018). As a skill-dominant combat sport, boxers operate in constantly evolving environments, requiring them to make decisions and counter based on opponents’ techniques and tactical maneuvers (Fiorilli et al., 2017; Liefeith et al., 2018; Çakmakçı et al., 2019). This supports conceptualizing agility as both physical and perceptual-cognitive. Given boxing’s emphasis—delivering clean, effective punches while avoiding opponent strikes (Guidetti, Musulin & Baldari, 2002)—athletes must strike precise blows to small target areas like the head and torso within the confined 6.1-m ring (Thomson & Lamb, 2016). This fundamentally demands exceptional agility to support rapid offensive-defensive reactions and movement (Chaabène et al., 2015; Slimani et al., 2017), further elevating the agility requirements for athletes. Therefore, executing critical boxing techniques necessitates exceptional agility and reaction speed. These attributes enable boxers to perform precise and rapid feints, footwork shifts, blocks, and abrupt changes in body position and direction (Sienkiewicz-Dianzenza & Maszczyk, 2019), thereby enhancing overall athletic performance.

Previous boxing research has primarily focused on tests related to strength, speed, and power (Di Prampero, Limas & Limas, 1970; Guidetti, Musulin & Baldari, 2002; Kravitz et al., 2003; Marković, Mišigoj-Duraković & Trninić, 2005; Popadic Gacesa, Barak & Grujic, 2009; Asok, 2010; Arseneau, Mekary & Léger, 2011; Hübner-Woźniak, Kosmol & Błachnio, 2011; Davis, Leithäuser & Beneke, 2014; Davis et al., 2015; Kim, Song & Min, 2016; Thomson & Lamb, 2016; Slimani et al., 2017; Bruzas et al., 2018). Agility tests crucial for boxing lack specificity to the sport, such as using 1-min hexagonal jumps and 3-min double-under skipping rope (Wu et al., 2024a; Niu et al., 2024); or categorizing agility into the ability to alter movement (continuous dodging defense), the ability to change direction (1-min four-quadrant jumps), coordination and balance (5 m walking forward after rotation) (Zhong & Bu, 2022), or repeated short-distance sprints (shuttle run 4 × 10 m with carrying blocks) (Sienkiewicz-Dianzenza & Maszczyk, 2019) to assess boxing-specific agility. However, these primarily change-of-direction tests do not fully replicate the demands of the competitive environment (Young, Dawson & Henry, 2015). Agility encompasses not only directional or velocity changes but also the perceptual-cognitive decision-making process and its outcome (Brughelli et al., 2008). In particular, when evaluating the agility of boxers, in addition to their ability to change direction, factors such as perception and decision-making during matches, anticipation of opponents’ movements, and reaction speed should also be included. Only then can a more comprehensive assessment of a boxer’s agility be achieved. Some scientists argue that agility assessments lacking a reactive perceptual decision-making component fall outside the true scope of agility testing (Paul, Gabbett & Nassis, 2016). In other combat sports, such as taekwondo and karate, research has already been conducted on the development of sport-specific agility measures and their reliability and validity; these studies confirm that sport-specific agility measures more accurately reflect athletes’ agility (Chaabene et al., 2018; Ben Hassen et al., 2022; Hassen et al., 2025). Therefore, to optimize boxing training programs, enhance agility training effectiveness, and ultimately improve performance, it is vital to develop tests that simulate these specific demands (Sheppard & Young, 2006). Consequently, this study proposes a Boxing-Specific Agility Test (BSAT) and examines its reliability and validity. The study also aims to determine the relationship between the BSAT and planned agility, perceptual decision-making, sprint time, jumping ability, and eye-hand coordination. The hypothesis is that the BSAT will demonstrate high reliability and validity, and show significant associations with other physical performance indicators relevant to boxing success.

## Materials and Methods

### Experimental approach to the problem

**Figure 1:**
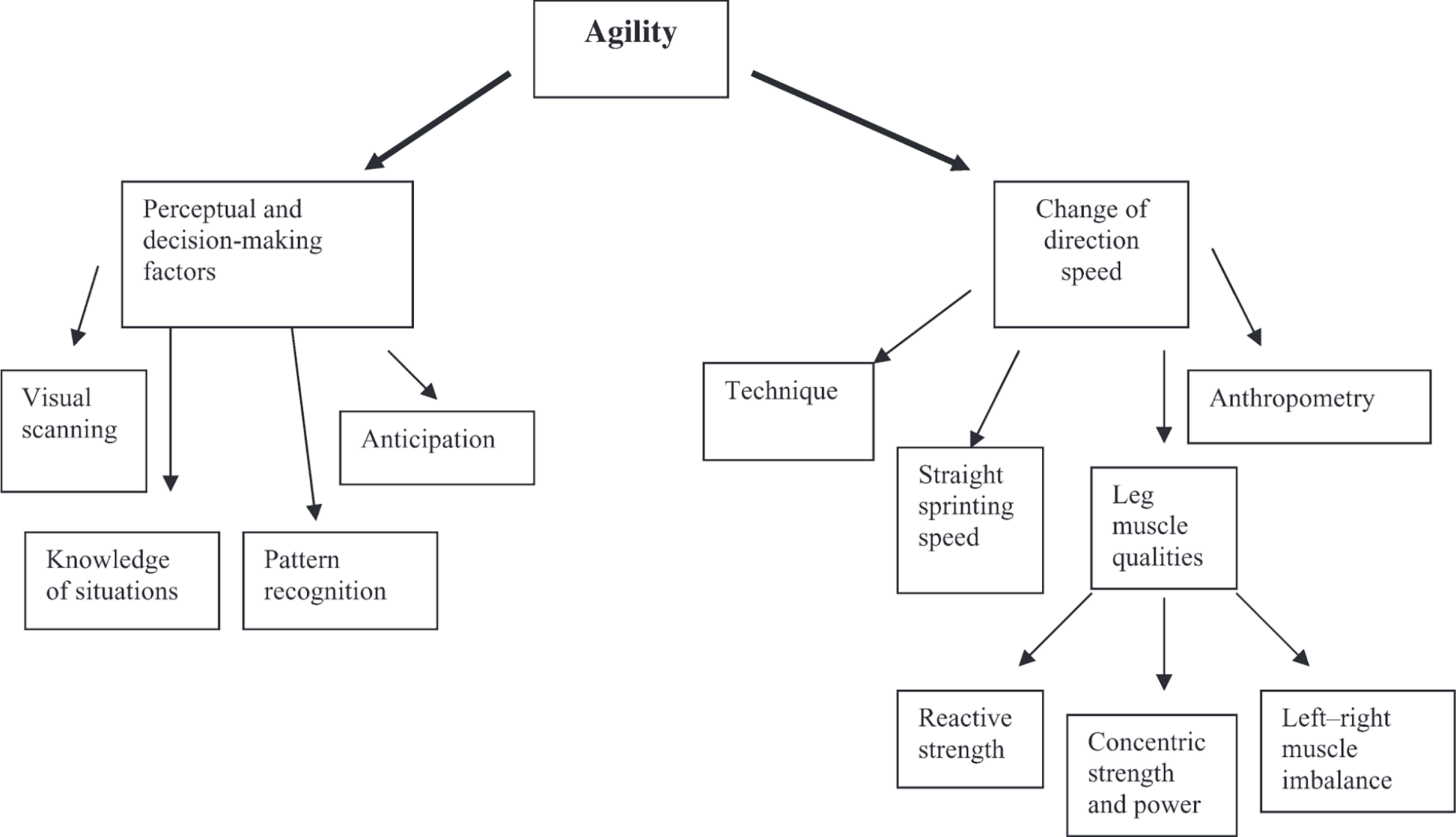
Agility components.

To execute the study aim, boxers completed the BSAT on two occasions separated by 7 days (During these 7 days, all participants underwent exactly the same training regimen under identical conditions, avoided high-intensity training, and followed a standard diet plan) to determine the reliability of the test (Chaabene et al., 2018; Putro et al., 2025). In addition, considering that agility also involves visual scanning, situational awareness, decision-making, linear sprint speed, and lower limb muscle quality (power) (Sheppard & Young, 2006) (see Fig. 1), tests of planned agility (T-test, Y pre-planned agility), reactive agility (Y reactive agility) (Fiorilli et al., 2017), perceptual decision-making, sprint performance (6 and 10 m) (Vänttinen, Blomqvist & Häkkinen, 2010; Krolo et al., 2020), lower body power (Counter movement jump (CMJ) and Squat-Jump (SJ)) (Sheppard & Young, 2006; Chaabene et al., 2018), and eye-hand coordination were completed (Both tests were conducted under exactly the same conditions and supervised by the same team). Testing was performed at the same of day in a boxing gym. Before all sessions, participants were hydrated and refrained from high-intensity physical activity for 48 h. Data were collected in April 2025 at the Boxing Management Center, Sports Bureau of the XinJiang Uygur Autonomous Region, China.

### Participants

Twenty-six elite adult male boxers (16 Chinese, 10 Cuban) participated. All had competed in national and international events across various weight classes, including five world top-3 finishers, seven continental top-3 finishers (two Asian champions), and 14 national top-3 finishers (including seven national champions). All participants met the inclusion and exclusion criteria: they were in good health with no hospitalizations due to illness in the past 3 years; no history of injury or illness in the past 6 months; no history of being knocked out in official competitions or concussions in the past year; vision tested on the international standard chart within the range of 1.0–1.5, and no history of wearing corrective lenses for myopia. Testing occurred during the general preparation phase of their annual competition cycle; therefore, no participants were undergoing specific weight-cutting procedures. All participants underwent training content and loads arranged and supervised by the same coaching team. To ensure participants were not affected by fatigue or unintentional weight reduction during testing, no high-intensity training was scheduled in the 2 days preceding the test. Based on international/national competition results and performance, they were divided into the Top Elite Group (*n* = 12; ≥ continental top-3; five Cubans, seven Chinese; Age: 22.75 ± 4.09 years; Training: 11.92 ± 1.68 years; Height: 182.33 ± 8.88 cm; Weight: 76.25 ± 18.14 kg) and the General Elite Group (*n* = 14; five Cubans, nine Chinese; Age: 21.36 ± 2.44 years; Training: 11.21 ± 2.81 years; Height: 185.5 ± 5.59 cm; Weight: 83.43 ± 13.44 kg). The grouping design in this study drew upon prior research on the reliability and validity of planned specialized agility trials in other combat sports (*e.g*., taekwondo), employing a pre-established grouping scheme (Chaabene et al., 2018). Results showed no differences (*p* > 0.05) in demographic traits between groups. The study was conducted according to the Declaration of Helsinki and approved by the Shanghai University of Sport Research Ethics Committee (Code: 102772024RT049, Approval date: 2024/05/22). All participants provided written informed consent before participating in the study.

### Overview of procedure

#### Familiarization session

Two familiarization sessions were conducted 1 week before formal testing to ensure athletes understood the procedures. To avoid disrupting athletes’ regular training, the two familiarization tests scheduled 1 week prior to the main testing were conducted during Wednesday afternoon’s recovery training session and following Saturday afternoon’s practical training session. The specific arrangement was as follows: During Wednesday’s recovery training session, researchers first explained the BSAT to participants, clarifying the test’s purpose and importance while outlining the specific testing procedures. Athletes then sequentially performed 2–3 preliminary tests, which were not counted towards final scores. During these sessions, researchers identified, documented, and pointed out any non-standard movements observed in athletes’ test execution, instructing them to correct these immediately to prevent recurrence during the formal test. Finally, researchers verbally confirmed with all participants their familiarity with the BSAT testing procedures and standards to ensure smooth, standardized execution during the formal test and obtain valid results. Testing on Saturday afternoon included: After athletes from each group complete their subsequent training following their sparring session, researchers conducted relevant tests (*e.g*., sprint runs, CMJ, *etc*.). Following their stretching, researchers conducted at least two pre-tests per athlete, not counting toward final scores. During these sessions, researchers promptly documented and addressed any issues observed to prevent recurrence in the formal testing. Following these tests, the athletes who had completed the preliminary tests were led by the experimenters to the laboratory for the preliminary eye-hand coordination test. Following the same procedure as above, the athletes underwent one preliminary test after fully understanding the eye-hand coordination testing process. Finally, the researchers verbally confirmed whether the athletes clearly understood all test procedures to ensure the formal test proceeded normally and the test results were valid.

#### Testing session

In all the tests where athletes were required to sprint, the timing gates (Fusion Sport) equipment was used for recording. Prior to testing, athletes uniformly performed a 15-min standardized warm-up under coach guidance (comprising: 3 min of slow jogging, 7 min of dynamic stretching, 2 min of jump rope, and 3 min of shadow punching). The BSAT was conducted on a standard boxing ring. The 6 and 10 m sprints, CMJ, SJ, T-test, Y pre-planned agility test, and Y reactive agility tests were performed on the training floor of the indoor boxing gym. These tests took place on Saturday afternoon from 3:00 PM to 6:00 PM. To minimize fatigue’s impact on the BSAT test and account for cumulative fatigue across different events, the testing sequence was: BSAT, 6 m sprint, 10 m sprint, CMJ, SJ, T-test, Y pre-planned agility test, and Y reactive agility tests. To prevent learning effects during testing, all participants completed one trial of each test in ascending order of body weight (weight was used as the testing sequence criterion to maximize consistency in rest intervals between participants). A second trial was then conducted for each test. This approach also minimized fatigue’s impact on performance. Given the varying durations of each test, a minimum 3-min rest interval was maintained between each trial. A 10-min rest interval was maintained between all tests, with individual rest intervals varying by no more than 2 min per participant. The eye-hand coordination test was conducted using the Senaptec Sensory Station in a darkened room with curtains closed, eliminating all sunlight and internal lighting. Due to the extended duration of each session, testing was divided into two blocks (Sunday 9:00 AM–12:00 PM and 3:00 PM–6:00 PM). Each unit had half the participants tested in the aforementioned sequence. Specific start times were assigned to each participant, who arrived at the testing site 5 min early to prepare (no warm-up required, but were required to be at least 1 h after waking).

### Assessment of physical fitness variables

*Boxing-Specific Agility Test (BSAT):*

**Figure 2:**
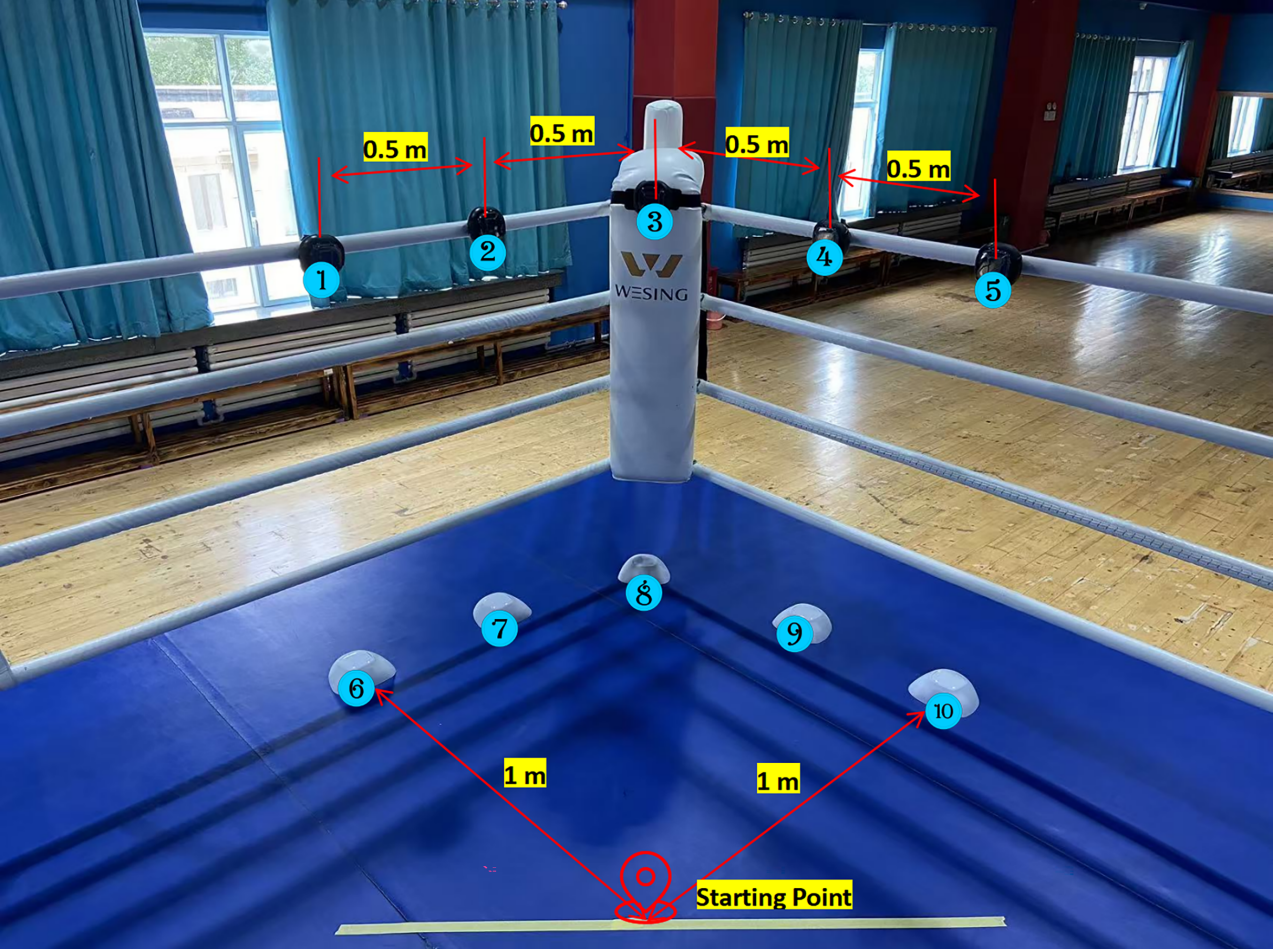
Diagram of BSAT.

**Figure 3:**
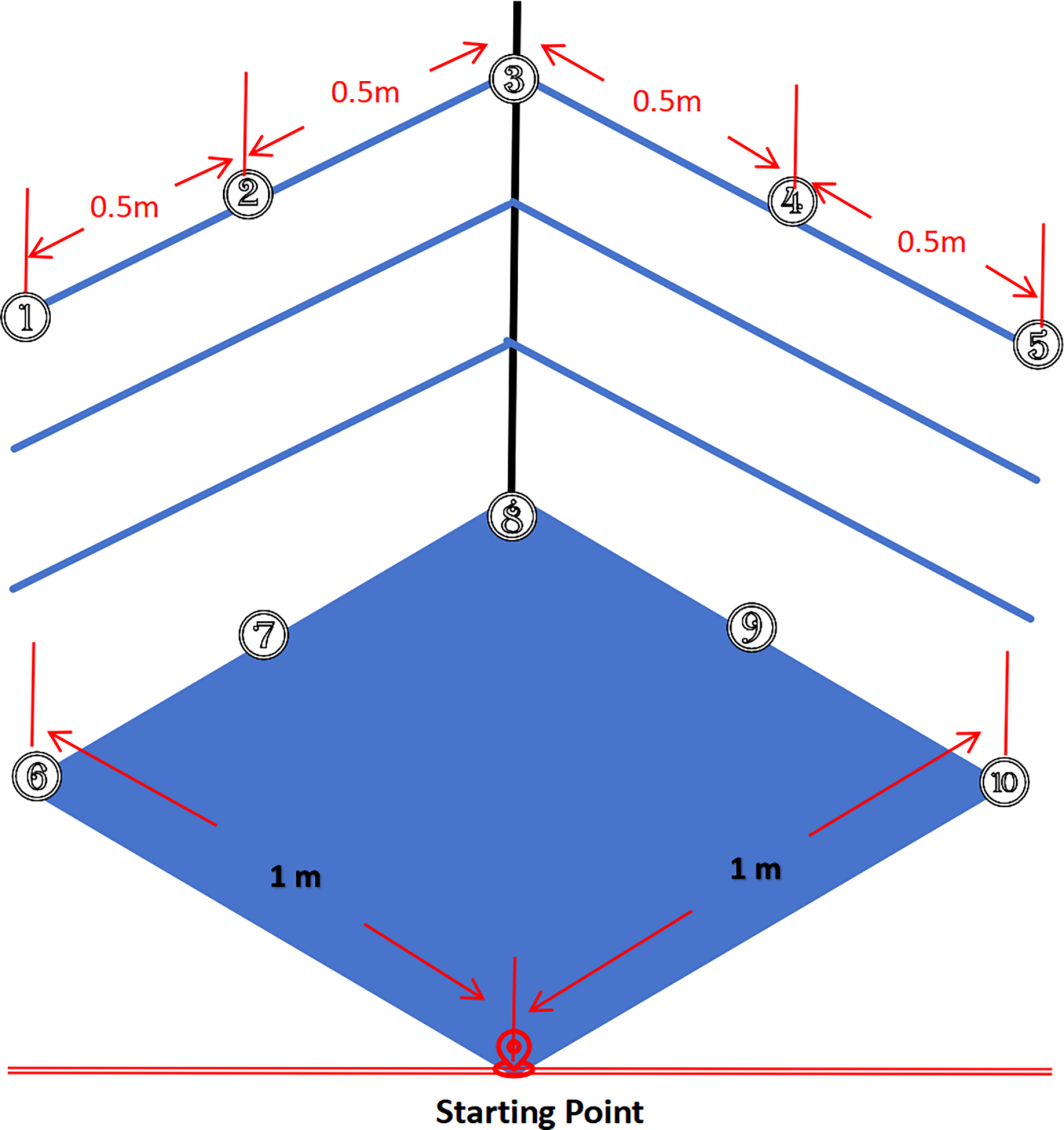
A simplified diagram of BSAT.

Based on the rules and characteristics of amateur boxing, and drawing on the “Stop’n’go” reaction time test (Sekulic et al., 2014), the BSAT was developed (see Figs. 2 and 3). Utilized the A-champs ROXs system. The visual stimulation system of this device primarily employs signal lights flashing different colors to present visual cues. To ensure the ecological validity of the BSAT and maximize alignment with official competition standards, athletes wore gloves of the corresponding weight class as per regulations (10 oz for 48–67 kg weight classes; 71–92+kg divisions wore 12 oz gloves), boxing shoes (meeting competition standards), and mouthguards (meeting competition standards). Athletes assumed their competition stance as the starting position (toes not crossing the starting line) and maintained that stance throughout the entire test. To enhance test-retest reliability, each athlete used the exact same set of equipment for both test attempts. Specific procedure: (a) The participant first visually scanned which of the 10 signal lights displayed a green light (the remaining nine signal lights remained transparent and unchanged), then swiftly moved toward the green signal using boxing-specific shuffling footwork. Participants struck the green signal with the knuckles of their gloved fist (if the green signal was one of the top five signals, *i.e*., signals 1–5) or lightly touched the top of the bottom five signals (signals 6–10) with the toe of either their front or back foot. (b) Upon touching the green traffic light and confirming it has returned to transparent (colorless), the athlete slid back to the start position (both feet behind the line). (c) Steps (a) and (b) were repeated for a total of 15 randomly cued light stimuli (The sequence of light stimulation was completely randomized according to the software algorithm built into the A-champs ROXs). Timing started upon remote initiation and stopped when the last light was struck/touched. (d) Outcome measures were total test time (s) and average response time (s) (The timing program of the test system had an accuracy of one-hundredth of a second). Three trials were performed with a minimum 3-min rest period between each, and the best time was used for analysis.

*Planned Agility Test (T-Test & Y Pre-Planned Agility):*

*T-Test*: Following established protocols (Hachana et al., 2013; Dunn et al., 2017) and its application in boxing (Sienkiewicz-Dianzenza & Maszczyk, 2019), four cones were arranged in a T shape: Cone A (start), Cone B 9.14 m ahead, Cone C 4.57 m left of B, Cone D 4.57 m right of B. From behind the start line, participants: sprinted forward to touch Cone B (right hand), shuffled left (facing forward) to touch Cone C (left hand), shuffled right (facing forward) to touch Cone D (right hand), shuffled left back to touch Cone B (left hand), then ran backwards to the start/finish. Trials were invalidated if cones were touched incorrectly, non-shuffling steps were used for lateral movement, or forward facing was not maintained. The testing process was strictly monitored by multiple researchers. Should any test prove invalid, retesting occurred after all participants had completed the test. If multiple retests were required, a minimum 3-min interval was maintained between tests. The best time of the three trials (3 min rest) was used. Previous T-Test test-retest Intraclass Correlation Coefficients (ICCs) range from 0.83–0.95 (Pauole et al., 2000; Vänttinen, Blomqvist & Häkkinen, 2010; Krolo et al., 2020); ICC in this study was 0.943.

*Y Pre-Planned Agility* (Oliver & Meyers, 2009; Fiorilli et al., 2017): Participants sprinted 5 m straight from the start gate. At the 5 m point, they pre-determinedly turned 45° left or right and sprinted another 5 m through the finish gate. The best time of the three trials (pre-determined direction, 3 min rest) was used. Test-retest ICC was 0.857.

*Reactive Agility Test (Y Reactive Agility) & Perceptual Decision-Making Time:*

*Y Reactive Agility* (Oliver & Meyers, 2009; Fiorilli et al., 2017): In this test, participants were unaware before the start whether the change of direction after the 5-m sprint would be to the left 45 degrees or right 45 degrees. After the participant sprints past the 2.5-m light source (transparent sensor light) at the starting point, a randomly selected indicator light—either on the left or right side ahead—illuminated with a steady green flash. The participant made a quick and accurate judgment, then changed direction and sprinted toward the illuminated light to complete the test at the finish timing gate on that side. The best time of the three trials (3 min rest) was used. Test-retest ICC was 0.872.

*Perceptual Decision-Making Time*: Calculated as the difference between the best Y Reactive Agility time and the best Y Pre-Planned Agility time (Y-REAC minus Y-PLAN), termed REAC-INDEX (Fiorilli et al., 2017). Test-retest ICC for decision-making time (DT) was 0.87.

*Lower Limb Power Tests (CMJ & SJ)*: Countermovement Jump (CMJ) and Squat Jump (SJ) were established measures of lower limb power in boxing research (Pauole et al., 2000; Sporis et al., 2010; Munro & Herrington, 2011; Stewart, Turner & Miller, 2014; Sekulic et al., 2017). For CMJ, starting upright with hands on hips, participants performed a rapid downward countermovement (flexing hips, knees, ankles) followed immediately by maximal vertical extension. For SJ, starting stationary in a self-selected semi-squat position (knees ~90°) with hands on hips, participants jumped vertically maximally without a preparatory countermovement. Jump height (cm) was measured using a contact mat (Smart Jump). The best height of three trials (3 min rest) was used for each test. Test-retest ICCs were CMJ: 0.965, SJ: 0.957.

*Sprint Tests* (6 & 10 m): Participants started from a standing position 0.3 m behind the first gate (height 0.75 m), initiating movement voluntarily. Time started when the first gate beam was broken and stopped when the second gate beam (at 6 or 10 m) was broken. The best time of the three trials (3 min rest) was used. Test-retest ICCs were 6 m: 0.862, 10 m: 0.910.

*Eye-hand Coordination (EHC)* Test: eye-hand coordination is strongly related to punching performance in boxing (Wu et al., 2024b), This study used the validated Senaptec Sensory Station System (Measurement accuracy was at the millisecond level) (Appelbaum & Erickson, 2018; Poltavski, Biberdorf & Praus Poltavski, 2021). Before each test unit commenced, the testing equipment underwent standardized calibration. Screen brightness, height, and other parameters were adjusted according to the operating manual, ensuring the testing environment remained free from external interference. Participants stood ~0.5 m from the screen (adjusted for height/reach). The test displayed a grid of 80 equally spaced, bordered circles (10 columns × 8 rows). One circle turned green at random; participants rapidly touched it with either index finger. Upon correct touch, it reverted to blank, and another circle turned green. This continued for 80 touches (The generation of lighting sequences was based on algorithms within the device system). Total time (ms) and average time per touch (ms) were recorded. Due to task specificity, repeated testing was not feasible within a short period. Test-retest ICCs were EHC Total Time: 0.869, EHC Average Time: 0.844.

### Statistical analyses

All analyses were performed using SPSS 27.0 (SPSS, Inc., Chicago, IL, USA). Data are presented as Mean ± Standard Deviation (SD). Normality was assessed using the Shapiro-Wilk test. Paired sample t-tests were used to determine learning effects or systematic bias between BSAT test and retest scores. Independent sample t-tests were used to compare the Top Elite and General Elite groups. Relative reliability was assessed using Intraclass Correlation Coefficients (ICC; two-way mixed-effects, absolute agreement, single measurement). Absolute reliability was expressed as the Typical Error of Measurement (TEM) and Coefficient of Variation (CV). TEM was calculated as TEM = SD_diff/√2 (where SD_diff is the SD of the differences between test-retest best scores). CV (%) was calculated as CV = (TEM/overall mean) × 100. Test usefulness was evaluated by comparing the Smallest Worthwhile Change (SWC) to TEM. SWC was assumed as 0.2 × between-participant SD (SWC0.2, small effect) (Hopkins et al., 2009) or 0.6 × SD (SWC0.6, moderate effect) (Hopkins, 2006). Test sensitivity was rated “Good” if TEM < SWC, “OK” if TEM ≈ SWC, or “Marginal” if TEM > SWC (Hopkins, 2004). The Minimal Detectable Change at 95% confidence (MDC95%) for the BSAT, representing the threshold for a real change beyond measurement error, was calculated as MDC95% = TEM × 1.96 × √2 (Haley & Fragala-Pinkham, 2006). Effect sizes for independent t-tests (Cohen’s d) were interpreted as small (0.00 ≤ d < 0.50), medium (0.50 ≤ d < 0.80), or large (d ≥ 0.80) (Cohen, 2009). Discriminant validity (ability to distinguish Top Elite from General Elite) was established *via* Receiver Operating Characteristic (ROC) curve analysis (Chaabène et al., 2012). BSAT best time was the test variable; group (Top Elite = 1, General Elite = 2) was the state variable. An Area Under the Curve (AUC) > 0.70 indicates good discriminant validity (Deyo & Centor, 1986). Pearson or Spearman correlation coefficients (based on normality) assessed relationships between BSAT times and T-test, CMJ, SJ, Y pre-planned agility, Y reactive agility, decision-making time, 6 m sprint, 10 m sprint, and EHC times. Effect sizes for correlations were qualitatively assessed: trivial r < 0.1, small 0.1 ≤ r < 0.3, moderate 0.3 ≤ r < 0.5, strong 0.5 ≤ r < 0.7, very strong 0.7 ≤ r < 0.9, nearly perfect r ≥ 0.9, perfect r = 1 (“New View of Statistics: Effect Magnitudes”). The coefficient of determination (R²) was calculated to determine shared variance (R² = r² × 100%). Statistical significance was set at *p* < 0.05.

## Results

### Reliability assessment

Paired sample t-tests revealed no significant differences between test and retest performances for BSAT Total Time (*p* = 0.13) or Average Time (*p* = 0.18), indicating no systematic learning effect. As presented in Table 1, the BSAT demonstrated excellent test-retest reliability with ICC values exceeding 0.96 and coefficients of variation below 4% for both measures. The TEM values were lower than the SWC0.2, confirming good sensitivity for detecting meaningful performance changes.

**Table 1:** Test–retest reliability of the boxing-specific agility test (BSAT).

Measure	ICC (95% CI)	TEM (s)	TEM (%)	SWC0.2 (s)	SWC0.6 (s)	MDC95% (s)
BSAT total time	0.966 [0.925–0.985]	0.92	2.61	1.04	3.11	2.55
BSAT avg. time	0.966 [0.927–0.985]	0.06	3.43	0.07	0.21	0.17

**Note:**

BSAT, Boxing-Specific Agility Test; ICC, intraclass correlation coefficient; CI, confidence interval; TEM, typical error of measurement; SWC, smallest worthwhile change; MDC95%, The threshold for determining real performance change (95% confidence). Changes exceeding these values reflect genuine improvements.

### Discriminant validity

The Top Elite group significantly outperformed the General Elite group in both BSAT Total Time (30.85 ± 3.52 s *vs*. 37.97 ± 3.39 s; *p* < 0.001) and Average Time (1.5 ± 0.23 s *vs*. 1.98 ± 0.23 s; *p* < 0.001), with large effect sizes (d > 2.0). ROC analysis yielded an AUC of 0.93 (95% CI [0.84–0.99]; *p* < 0.001), confirming strong discriminant validity for distinguishing between competitive levels.

### Criterion and construct validity

**Figure 4:**
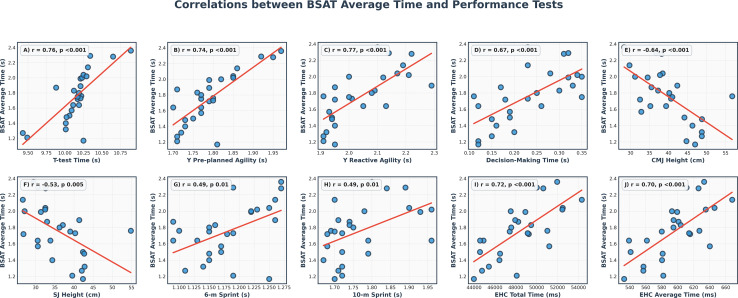
XY scatter chart of BSAT avg time.

**Figure 5:**
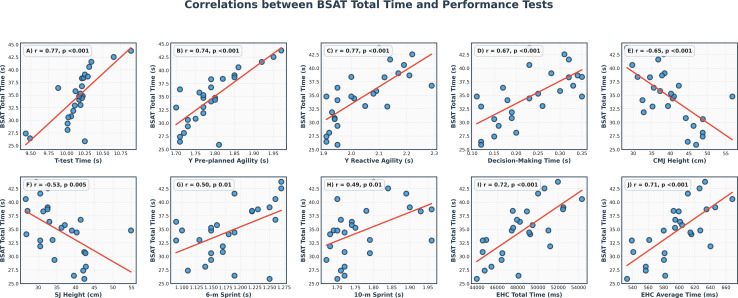
XY scatter chart of BSAT total time.

Significant correlations were found between BSAT and other tests (Table 2, Figs. 4 and 5). Both BSAT Total Time and Average Time showed very strong correlations with the T-test (r = 0.77, r² = 59.2%, *p* < 0.001; r = 0.76, r² = 57.7%, *p* < 0.001) and Y-reactive agility (r = 0.77, r² = 59.2%, *p* < 0.001; r=0.77, r² = 59.2%, *p* < 0.001). The correlations with EHC Total Time and Average Time were also very strong (r = 0.71–0.72, p < 0.001). BSAT times demonstrated strong correlations with lower body power jumps (r = 0.58–0.62, *p* < 0.01) and moderate correlations with sprint times (r = 0.49–0.50, *p* < 0.05).

**Table 2:** Correlations of BSAT with performance tests (r and *p*-values).

		BSAT total time		BSAT avg. time	
Test	Mean ± SD	r (95%CI)	*p*	r (95%CI)	*p*
T-Test (s)	10.15 ± 0.28	0.77 [0.53–0.89]	<0.001	0.76 [0.53–0.89]	<0.001
YPA (s)	1.79 ± 0.73	0.74 [0.49–0.89]	<0.001	0.74 [0.48–0.88]	<0.001
YRA (s)	2.04 ± 0.11	0.77 [0.54–0.90]	<0.001	0.77 [0.54–0.90]	<0.001
DMT (s)	0.23 ± 0.08	0.67 [0.37–0.84]	<0.001	0.67 [0.37–0.84]	<0.001
CMJ (cm)	39.89 ± 6.84	−0.65 [−0.33 to −0.82]	<0.001	−0.64 [−0.33 to −0.82]	<0.001
SJ (cm)	35.86 ± 6.59	−0.53 [−0.17 to −0.76]	0.005	−0.53 [−0.17 to −0.76]	0.005
6-m sprint (s)	1.18 ± 0.06	0.50 [0.13–0.74]	0.01	0.49 [0.12–0.73]	0.01
10-m sprint (s)	1.77 ± 0.09	0.49 [0.11–0.74]	0.01	0.49 [0.11–0.74]	0.01
EHC total time (ms)	48,414.58 ± 2,825.58	0.72 [0.46–0.86]	<0.001	0.72 [0.45–0.86]	<0.001
EHC avg. time (ms)	596.69 ± 35.10	0.71 [0.43–0.86]	<0.001	0.70 [0.42–0.85]	<0.001

**Note:**

CI, confidence interval; BSAT, Boxing-Specific Agility Test; YPA, Y Pre-Planned Agility Test; YRA, Y Reactive Agility Test; DMT, Decision-Making Time; CMJ, Counter Movement Jump; SJ, Squat jump; EHC, Eye-Hand coordination.

## Discussion

This study is the first to develop and validate a specific agility test for amateur (Olympic) boxing. Key findings were: (a) The BSAT showed no learning effect in test-retest analysis, demonstrating extremely high reliability; (b) both BSAT Total Time and Average Time clearly distinguished between Top Elite and General Elite athletes; (c) the BSAT showed very strong correlations with the T-test (gold standard for planned agility) and the Y pre-planned agility test, and also with the Y reactive agility test which incorporates reaction and decision-making; (d) BSAT times showed very strong correlations with both EHC Total Time and Average Time; (e) additionally, BSAT times demonstrated moderate to strong correlations with other tests like lower body power jumps and sprint times.

The BSAT test-retest ICC values (>0.90 for both measures; ICC = 0.966) indicate exceptional reliability, exceeding that typically reported for the T-test (ICC = 0.83–0.95 (Pauole et al., 2000; Vänttinen, Blomqvist & Häkkinen, 2010; Krolo et al., 2020)). Furthermore, low CV% values (<5%; Total Time 2.61%, Average Time 3.43%) confirm the BSAT’s reliability. The TEM values were lower than SWC0.2, indicating the BSAT is highly sensitive for detecting small, meaningful changes in performance, aligning with findings from a specific agility test in taekwondo (Chaabene et al., 2018). The MDC95% defines the threshold for a physiologically meaningful and performance-relevant improvement beyond random variation (Mann et al., 2016). The MDC95% values (Total Time 2.55 s, Average Time 0.17 s) indicate that improvements of this magnitude represent genuine performance gains in elite boxers.

Independent t-tests confirmed that Top Elite athletes significantly outperformed General Elite athletes on the BSAT. ROC analysis further validated the BSAT’s discriminant validity for distinguishing agility levels between competitive tiers. The BSAT showed very strong correlations with traditional planned agility tests (T-test and Y pre-planned agility), sharing nearly 60% variance with the T-test “gold standard.” Although the T-test primarily assesses change-of-direction speed (Stewart, Turner & Miller, 2014; Mann et al., 2016), its widespread use for agility assessment supports the validity of the BSAT as a specific tool for evaluating agility in boxers.

This study also assessed Y reactive agility, which incorporates reaction and perceptual decision-making into the Y-plan format, aligning with the expert definition of agility (Sheppard & Young, 2006), and its association with strength, speed, cognition, and decision-making (Fiorilli et al., 2017; Liefeith et al., 2018). The derived perceptual decision-making time (DT) is highly correlated with agility (Morral-Yepes et al., 2022), and the cognitive component is vital for performance (Young, Dawson & Henry, 2015). Reactive and decision-making abilities are crucial for agility (Inglis & Bird, 2016), and are particularly important in boxing (Yordanova et al., 2023), with faster reaction times and better decision-making linked to superior performance (Wu et al., 2024b). Overall, in the Y Reactive Agility test, athletes need to rapidly assess their next directional move within an extremely brief timeframe. This ability shares similar physiological factors with the BSAT, which requires athletes to quickly identify which light illuminates and make decisions accordingly, as well as with boxing scenarios where athletes must assess and decide their next action based on an opponent’s varied behavioral responses. The strong correlations found between Y-reactive agility, DT, and the BSAT support these findings.

CMJ and SJ performances were consistent with previous literature on elite boxers (Sporis et al., 2010; Munro & Herrington, 2011; Stewart, Turner & Miller, 2014; Sekulic et al., 2017). Lower limb power is a key component of boxing (Walilko, Viano & Bir, 2005), contributing to punch impact, speed, and agility performance (Kim, Lee & Park, 2018), and is included in physical performance assessment models for male boxers (Bu, 2022; Wu et al., 2024a). The strong correlations between BSAT times (Total time and Avg. Time) and CMJ/SJ indicate that lower limb power (both stretch-shortening cycle and concentric) is associated with agility in boxers. The triple flexion/extension of the hip, knee, and ankle during agility movements relates to jumping mechanics (Zhou et al., 2022; Chen et al., 2023), and jumping ability/vertical force production likely enhances agility (Brughelli et al., 2008). The lower-body kinetic chain utilized in lower-body explosive power testing exhibits a high degree of similarity to the shuffling movements performed by athletes during BSAT and boxing competitions. In official competition, every footwork movement by a boxer needed to be rapid and highly explosive while simultaneously generating power for punching. Therefore, the significant correlation observed in this study between BSAT total time and average time with lower-body explosive power tests (CMJ and SJ) aligns with the characteristics and physiological demands of boxing.

The BSAT showed moderate correlations with 6 and 10 m sprint times, though weaker than other tests. This may relate to several factors: the confined space of the boxing ring (6.1 × 6.1 m), the daily training habits of boxers, the technical requirements of competition (which do not involve sprinting movements), and the athletes’ technical and tactical limitations. Additionally, pure linear sprinting is less prominent than agility during bouts. However, sprinting speed is a component of agility (Brughelli et al., 2008; Mann et al., 2016), has been used in boxing agility/performance assessment models (Bu, 2022; Zhong & Bu, 2022; Wu et al., 2024a; Niu et al., 2024), and the moderate-to-strong correlations (r = 0.49–0.50, R² ≈ 24–25%) indicate a significant association between BSAT performance and boxers’ short-distance acceleration capabilities. Previous studies have also indicated that athletes’ sprinting ability correlates with factors such as lower-body explosive power and coordination (Villanueva-Guerrero et al., 2024). Since lower-body explosive power and coordination are key determinants of an athlete’s agility, it is reasonable that the BSAT test shows a moderate correlation with the sprinting ability of boxers.

The Senaptec EHC test results showed significant very strong correlations with BSAT times (R² ≈ 49–51.8%). This is expected, as the BSAT design inherently involves visual information acquisition, cognitive processing, and precise hand/foot motor responses, closely linking it to EHC. Literature supports the relationship between eye-hand coordination and agility (Wong et al., 2019), and its importance for boxing performance (Wu et al., 2024b; Mahlangu et al., 2024). Overall, the athletic performance of boxers is inseparable from their eye-hand coordination abilities, which is one of the core design principles behind BSAT. Since every movement in boxing matches relies on the coordinated action of eyes, hands, and feet, the findings of this study align with the practical demands of actual boxing competition.

Limitations: First, the sample in this study was limited to elite-level male boxers. Future research could expand to female boxers or adolescent boxers based on the methodology and experimental design proposed herein. Second, this study did not differentiate between athletes’ stances, specifically whether they were orthodox or southpaw. This is because the BSAT is designed as a symmetrical test, and theoretically, an athlete’s stance should not produce different test results. However, this has not been scientifically validated. Nevertheless, the results align with the number of participants meeting the inclusion and exclusion criteria. Given the limitations of this study, it was not feasible to further group or exclude participants based on stance differences. Therefore, we hope that future research will delve deeper into this aspect. Third, amateur boxing match consists of three 3-min rounds, totaling 9 min of competition. Boxers inevitably experience varying degrees of fatigue during the second and third rounds, and much of boxing is performed in a (pre-) fatigued state. However, fatigue simulation conditions were not established in this study. Therefore, subsequent studies should incorporate fatigue simulation protocols into their experimental designs. Additionally, due to the inability to standardize test sequences, content, and timing across participants, this study did not quantify or standardize fatigue factors during testing. Future research should address this limitation. Fourth, the BSAT test in this study utilized only time as a metric. However, indicators such as power output, reaction time, and hit rate are also critically important in boxing. Future research designs should therefore develop or utilize more specialized equipment to conduct comprehensive evaluations. Furthermore, it should be acknowledged that the correlations observed in this study do not imply causation, and the cross-sectional design precludes causal inferences about the relationships between variables.

## Conclusions

The Boxing-Specific Agility Test (BSAT) tested in this study is the first standardized assessment tool for specific agility in boxing. It has demonstrated excellent reliability, validity (construct, criterion, discriminant), and sensitivity for detecting meaningful changes, enabling precise evaluation of boxers’ specific agility. Critically, the BSAT integrates key boxing-specific elements—reaction, perceptual decision-making, and eye-hand coordination—which are vital for performance. Beyond assessment, the BSAT’s significant correlations with jumping ability, reaction, decision-making, and eye-hand coordination suggest its potential utility as a training modality to holistically develop these performance-linked capacities. While designed for elite athletes with one target light among nine distractors, the BSAT protocol is adaptable. For lower-level athletes or training progression, modifications could include: using only one green light (no distractors), using distractors of a single color, or reducing the number of target lights (*e.g*., five initially), gradually increasing complexity. This flexibility enhances the BSAT’s value for both evaluation and training in boxing.

## Supplemental Information

10.7717/peerj.21458/supp-1 Supplemental Information 1 Basic Information of Participants.

10.7717/peerj.21458/supp-2 Supplemental Information 2 First test of raw data.

10.7717/peerj.21458/supp-3 Supplemental Information 3 Second test of raw data.

## Additional Information and Declarations

### Data Availability

The following information was supplied regarding data availability:

The raw data are available in the Supplemental Files.

### Clinical Trial Registration

The following information was supplied regarding Clinical Trial registration:

102772024RT049.
